# Additions to the species of *Magnolia* L., sect. *Manglietiastrum* (Y.W.Law) Noot. (Magnoliaceae) in Vietnam

**DOI:** 10.3897/phytokeys.277.191131

**Published:** 2026-07-20

**Authors:** Nguyen Thi Ai Minh, Tran Thai Vinh, Truong Thi Lan Anh, Nguyen Hoang Nghia, Hoang Thanh Truong, Lao Duc Thuan, Tran Van Tien

**Affiliations:** 1 Dalat University, Dalat City, Lam Dong Province, Vietnam Faculty of Biotechnology, Ho Chi Minh City Open University Ho Chi Minh Vietnam; 2 Institute of Life Sciences, Vietnam Academy of Science and Technology, Dalat, Vietnam Dalat University Dalat Vietnam; 3 Vietnam Academy of Forest Science, Hanoi, Vietnam Vietnam Academy of Forest Science Hanoi Vietnam; 4 Vietnam Forest Science Institute of Central Highlands and South of Central Vietnam, Vietnam Academy of Forest Science, Dalat City, Lam Dong Province, Vietnam Vietnam Forest Science Institute of Central Highlands and South of Central Vietnam, Vietnam Academy of Forest Science Dalat Vietnam; 5 Faculty of Biotechnology, Ho Chi Minh City Open University, Ho Chi Minh City, Vietnam Institute of Life Sciences, Vietnam Academy of Science and Technology Dalat Vietnam

**Keywords:** Magnoliaceae, morphology, new species, phylogeny, Vietnam

## Abstract

A new species of *Magnolia* L., sect. *Manglietiastrum* (Y.W.Law) Noot. (Magnoliaceae), *Magnolia tienii*, is described and illustrated from Vietnam based on detailed morphological observations. The new species is morphologically allied to *M. praecalva* and *M. sinica*, but it is readily distinguished by having paired terminal flowers borne on the twigs; elliptic leaves, 9.5–10.5 × 3.0–3.5 cm, with an attenuate base and a slightly attenuate to obtuse apex; and 11 tepals per flower. Phylogenetic analysis based on *rbcL* sequences confirms the placement of *M. tienii* within sect. *Manglietiastrum*. The *rbcL* sequence differs by only a single nucleotide from those of its closest relatives, indicating a close relationship. These stable diagnostic characters strongly support the recognition of *M. tienii* as a distinct species.

## Introduction

*Manglietiastrum* (Y.W.Law) Noot. is a small section within *Magnolia* subgenus *Gynopodium* Figlar & Noot. and was established by Nooteboom in 1985. The section is characterized by stipules free from the petiole and lacking a stipular scar; solitary, bisexual flowers; nine tepals; a sessile gynoecium (although shortly stipitate in *Magnolia sinica* (Y.W.Law) Figlar & Noot.); carpels dehiscing mainly along the ventral suture, particularly near the apex of the fruiting body; and 3–8 ovules per carpel (Figlar and Nooteboom 2004). Comprising only three species, the section is narrowly distributed across Southeast Asia and Malesia. Among these, *Magnolia praecalva* (Dandy) Figlar & Noot., native to Penang (Malaysia) and Ba Na (Vietnam), was transferred from *Pachylarnax praecalva*Dandy (1927). It is characterized by elliptic-oblong to oblanceolate leaves with a cuneate to attenuate base and an obtuse to rounded apex, as well as more or less orbicular, loculicidal fruits prior to dehiscence. The second species, *Magnolia pleiocarpa* (Dandy) Figlar & Noot., from India, was transferred from *Pachylarnax pleiocarpa*Dandy (1933) and is distinguished by elliptic-oblong to oblanceolate-oblong leaves with a rounded to obtuse apex and similar suborbicular, loculicidal fruits. The third species, *Magnolia sinica* (Y.W.Law) Figlar & Noot., from China, was transferred from *Manglietiastrum sinicum* Y.W.Law (1979) and is characterized by narrowly obovate to narrowly obovate-elliptic leaves, nine tepals, and orbicular, loculicidal fruits. Several comprehensive studies of vegetative and reproductive characters have demonstrated that these traits can provide sufficient diagnostic characters for species identification.

**Figure 1.**
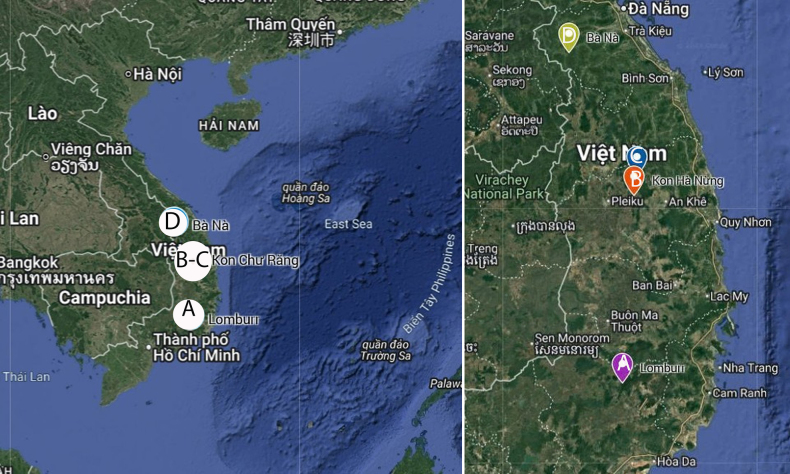
Distribution map. **A**. Lomburr; **B**. Kon Ha Nung; **C**. Kon Chu Rang; **D**. Ba Na. Source: Google Maps (2026).

**Figure 2.**
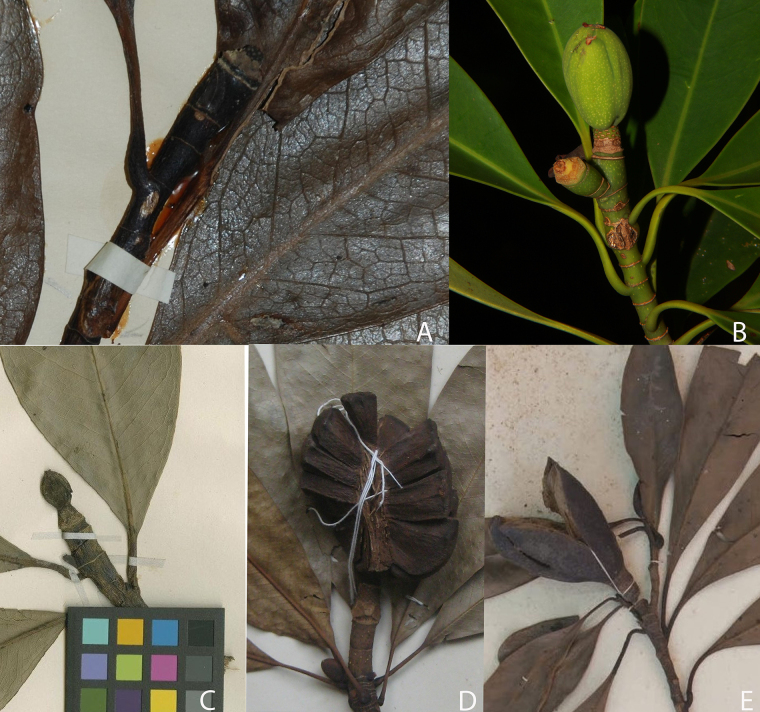
Peduncle nodes. **A**. *M. praecalva* (*Haniff 3485*, K); **B**. *M. tienii*; **C**. *M. pleiocarpa* (*Beat Officer 48427*, Herb. Brit. Mus.); **D**. *M. sinica* (0822509, KUN); **E**. *M. praecalva* (*Poilane 7264*, VNM00000678).

**Figure 3.**
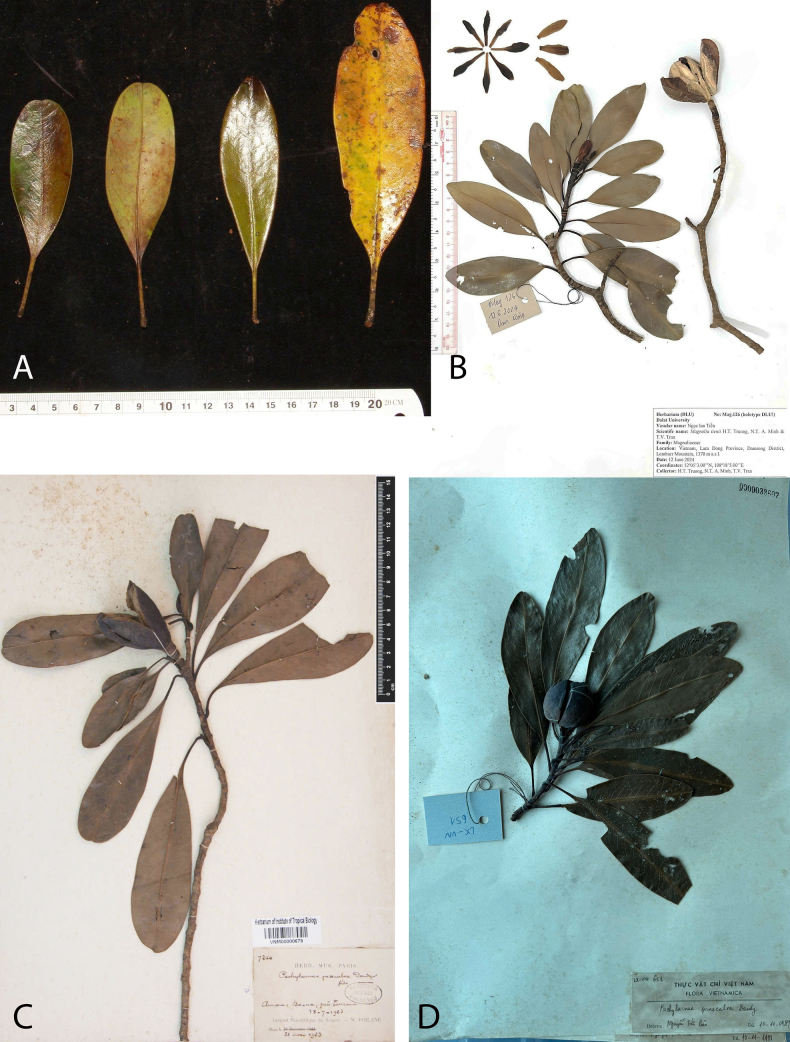
**A**. *DLUMag.131*; **B**. *DLUMag.126*; **C**. *Poilane 7264*; **D**. *Nguyen Tien Ban VN651*.

In 1927, Dandy established *Pachylarnax* as a new genus based on the type species *Pachylarnax praecalva*. Subsequently, Figlar and Nooteboom (2004) transferred the genus to *Magnolia*, placing it in *Magnolia* sect. *Manglietiastrum*. Dandy’s original description of *P. praecalva* was based on two specimens: flowering material from Malaysia deposited at K (Haniff 3485, 4067 [holotype]) and fruiting material bearing capsular fruits from Annam deposited at P (Poilane 7264). The originally described leaves were obovate to oblanceolate, with a cuneate base and an obtuse to rounded, often emarginate apex. Specifically, the K specimens (Haniff 3485 and 4067 [holotype]) exhibit elliptic-oblong to oblanceolate leaves, with a cuneate to attenuate base, a rounded apex, and peduncles with 3–4 nodes (Fig. 1). In contrast, the specimens deposited at P, VN, and VNM (*Poilane 7264*: P00203400, P00203401, P00203399; VNM00000676, VNM00000677, VNM00000678; *Nguyen Tien Ban VN651*: VN0000039607) possess obovate to oblanceolate leaves with a cuneate to attenuate base, an obtuse to rounded apex, and peduncles with only two nodes (Figs 2, 3). Thus, the literature and herbarium examinations indicate that leaf morphology exhibits substantial variation among regions, whereas the reproductive characters remain consistent, consisting of a solitary terminal flower with nine tepals (Dandy 1927; Sawangchote et al. 1999; Xia et al. 2008; Nooteboom 2012).

In June 2024, *Magnolia* specimens collected from Lomburr Mountain (Dam Rong District, Lam Dong Province, Vietnam) exhibited strong morphological affinities with *M. praecalva*, *M. pleiocarpa*, and *M. sinica*. To verify these findings, Vietnamese localities previously documented for *M. praecalva* based on herbarium records were revisited: Ba Na—the collection site of *Poilane 7264* (P00203400, P00203401, P00203399; VNM00000676, VNM00000677, VNM00000678)—and Kon Ha Nung—the site of *Nguyen Tien Ban VN651* (VN0000039607). Although no individuals were located at these historical sites, three individuals were discovered at Kon Chu Rang, approximately 50 km from the Kon Ha Nung location (Fig. 1), whose leaf morphology closely resembled that of the Ba Na specimens. A critical comparison with herbarium images indicates that the newly collected material from Lomburr Mountain is clearly distinct from all described species of *Magnolia* sect. *Manglietiastrum*. Although some of the new collections conformed to the diagnostic characters of *M. praecalva* (particularly in leaf arrangement), the unique combination of elliptic leaf morphology, paired terminal flowers, and 11 tepals supports recognition of the Lomburr Mountain population as a distinct species new to science.

The chloroplast *rbcL* gene (ribulose-1,5-bisphosphate carboxylase/oxygenase large subunit), which encodes the large subunit of the RuBisCO protein, is widely used as a robust marker in plant phylogenetics and evolutionary studies (Sheikina 2022; Candramila et al. 2023). It is frequently selected because of its relatively low mutation rate compared with other chloroplast DNA regions, enabling reliable phylogenetic inference at higher taxonomic levels (Harnelly et al. 2018; Sheikina 2022; Candramila et al. 2023). Furthermore, because of its conservative nature, it provides a stable baseline for comparative interspecific and phylogenetic analyses (Candramila et al. 2023). According to Kim and Suh (2013), the *rbcL* gene was included as one of 10 chloroplast DNA regions used for large-scale phylogenetic reconstruction within the family Magnoliaceae. In the present study, *rbcL* sequence data were used to confirm and resolve the phylogenetic position of the collected samples.

Because the collected specimens lacked flowers or fruits, detailed examinations of leaf morphology and anatomical traits were essential for their taxonomic evaluation (Tucker 1964; Hu et al. 2018). Previous research on the stomatal characteristics of Magnoliaceae has demonstrated that these characteristics possess significant taxonomic value (Tucker 1964; Baranova 1972; Hu et al. 2018). Consequently, in the current study, stomatal traits were also used to identify and differentiate the newly collected samples from closely related taxa.

## Materials and methods

### Plant materials

Plant material was collected from Lomburr Mountain (Dam Rong District, Lam Dong Province) and Kon Chu Rang (Gia Lai Province), located in the Central Highlands of Vietnam. Voucher specimens were deposited in the herbaria of Dalat University (DLU) and the Tay Nguyen Institute for Scientific Research (VTN) (Table 2).

### Morphological observation

Morphological characters, including leaves, flowers, and fruits, were dissected and examined, and vegetative traits were measured directly from fresh field material. Digital images of leaves from both field-collected and herbarium specimens (listed in Table 2) were obtained following the general approach of Igathinathane et al. (2008); however, conventional flatbed scanning was replaced by digital photography using a Canon EOS 600D camera. Morphometric measurements, including leaf length (cm), width (cm), and leaf area (cm^2^), were subsequently performed using ImageJ software (Abràmoff et al. 2004).

For all investigated plants, leaf epidermal traits were observed at identical positions on mature leaves, specifically on the third or fourth leaf from the terminal bud. Small leaf segments (1 × 0.5 cm) located near the midrib of fully expanded leaves were excised and fixed in 0.25% glutaraldehyde for at least 12 h. The fixed samples were then trimmed to 0.5 × 0.1 cm, rinsed three times in 0.1 M phosphate buffer for a total of 2 h, and dehydrated through a graded ethanol series of 30%, 50%, 70%, 80%, and 90% for 15 min each, followed by three changes of 100% ethanol and *tert*-butyl alcohol for 10 min each. Subsequently, the specimens were vacuum freeze-dried, mounted onto stubs using double-sided conductive tape, and sputter-coated with palladium–gold (Pd–Au). Micromorphological observations were conducted using a JEOL JSM-6360 LV scanning electron microscope (SEM) at an accelerating voltage of 20 keV. All morphological parameters were measured in quadruplicate using ImageJ software (Abràmoff et al. 2004).

The measured stomatal traits included stomatal density (no./mm^2^), stomatal length (SL, µm), and stomatal width (SW, µm) (Savvides et al. 2011). Rather than guard cell width, which exhibits high variability and a reduction of up to 50% upon stomatal closure, stomatal width was selected for measurement as a more stable parameter (Shope and Mott 2006). Differences in stomatal size among the specimens were analyzed using one-way analysis of variance (ANOVA), followed by Fisher’s least significant difference (LSD) post hoc test, performed using STATGRAPHICS Centurion XV version 15.1.02.

### Taxon sampling

Putatively related taxa were selected for morphological comparison based on original descriptions and subsequent taxonomic treatments of *Magnolia* sect. *Manglietiastrum* and allied groups (Dandy 1927, 1933; Nooteboom 1985, 2012; Sawangchote et al. 1999; Xia et al. 2008; Nooteboom and Chalermgling 2009). Diagnostic characters were evaluated through a detailed comparison of vegetative and reproductive traits, with particular emphasis on leaf shape, stomatal features, and floral structure.

### Specimens examined

The morphological features of the new species were verified by consulting specimens deposited in the herbaria K, P, VN, and VNM: *Haniff 4067* (K000681525), Peninsular Malaysia, Penang (holotype, K); *Haniff 3485*, Peninsular Malaysia, Penang; *Beat Officer 48427* (BM000551351), Lakhimpur, Assam, India (type, Herb. Brit. Mus.); *Poilane 7264* (P00203402); *Poilane 7264* (P00203399); *Poilane 7264* (P00203400); *Poilane 7264* (P00203401); *Poilane 7264* (VNM00000676); *Poilane 7264* (VNM00000677); *Poilane 7264* (VNM00000678); *Poilane 7264* (VNM00000679); *Nguyen Tien Ban VN651* (VN0000039607).

### DNA extraction, PCR assay, and sequencing

Genomic DNA was isolated from the samples using a modified phenol–chloroform method (pH 8.0). The samples were incubated overnight at 65 °C in a lysis buffer containing 2.0% SDS, Tris-HCl (pH 8.0), 150 mM NaCl, 10 mM EDTA, and 0.1 mg/mL proteinase K. Following incubation, the supernatant was recovered by centrifugation, supplemented with 700 μL of phenol/chloroform/isoamyl alcohol (25:24:1), and centrifuged again. The aqueous phase was then collected, and the DNA was precipitated using absolute isopropanol. Finally, the purified genomic DNA was eluted and stored in Tris-EDTA buffer at –20 °C for subsequent molecular analyses.

The sequences of the forward and reverse primers were ATGTCACCACAAACAGAGACTAAAGC and GTAAAATCAAGTCCACCRCG, respectively. PCR amplification was performed in a total volume of 15 μL under the following cycling conditions: initial denaturation at 95 °C for 5 min, followed by 40 cycles of denaturation at 95 °C for 30 s, annealing at 55 °C for 30 s, and elongation at 72 °C for 1 min, with a final extension at 72 °C for 5 min. Aliquots (5 μL) of the resulting PCR products were electrophoresed on a 2.0% agarose gel and visualized under a UV transilluminator. Finally, the amplified products were commercially sequenced by Nam Khoa Company (Vietnam).

### Taxa and *rbc*L reference sequence collection, phylogenetic analysis

A reference dataset for the *rbcL* gene was established by retrieving sequences from GenBank (NCBI) based on previous studies (Table 1). Each reference sequence was annotated with its respective accession number, taxon name, and geographic locality. The newly amplified DNA sequences were proofread and trimmed to remove ambiguous signals at both ends using SeaView version 4.2.12 and Chromas Lite version 2.1.1. Finally, phylogenetic reconstruction was performed using the maximum parsimony (MP) method in Molecular Evolutionary Genetics Analysis (MEGA) version X, with node support assessed using 1,000 bootstrap replicates.

**Table 1.** Representative taxon information and GenBank accession numbers for sequences used in the current study.

**No**.	**Taxon**	**Accession number**
1	*Magnolia kwangsiensis* voucher S. Kim 1053	MT682810
2	*Magnolia denudata* voucher BJ007	NC_056770
3	*Magnolia denudata*	JN867577
4	*Magnolia liliiflora*	JX280397
5	*Magnolia alba*	MF990568
6	*Magnolia liliiflora* voucher Chen 20110102073	MN990588
7	*Magnolia alba* voucher MALB20170517V5	NC_037005
8	*Magnolia denudata* voucher J. Wen 11333	MN990618
9	*Magnolia praecalva*	MT682876
10	*Magnolia sinica* voucher Chen 20110102045	MN990584
11	*Magnolia sinica*	OL631157
12	*Magnolia grandiflora* voucher Soltis and Miles 2764	MT682795
13	*Magnolia sprengeri*	JX280401
14	*Magnolia sprengeri* voucher Wilson 278	MN990634
15	Liriodendron chinense*	NC_030504
16	*Liriodendron chinense* voucher S. Kim 1044*	MT682877
17	*Liriodendron chinense* voucher J. Wen 11247*	MN990597
18	Liriodendron tulipifera*	MT682878
19	*Magnolia tienii* (1)	DLUMag.126.1
20	*Magnolia tienii* (2)	DLUMag.126.2
21	*Magnolia tienii* (3)	DLUMag.126.3

Note: *outgroup.

## Results

### Morphological comparison

Flowers and fruits

The most striking diagnostic feature of *M. tienii* is the presence of paired flowers borne terminally on the twigs, with each flower possessing 11 tepals. This unique combination of characteristics is absent in all allied species (Fig. 2).

#### Leaf sizes

The vegetative morphology is distinct, characterized by elliptic leaves with an attenuate base and a slightly attenuate to obtuse apex, measuring 9.5–10.6 × 3.0–3.5 cm (leaf area ca. 25.85 cm^2^). Morphological comparison among the specimens from Lomburr, Kon Chu Rang, Ba Na, Kon Ha Nung, and Peninsular Malaysia reveals that the Lomburr specimens possess the smallest leaves, whereas those from Peninsular Malaysia exhibit the largest. In addition, the leaf dimensions of the specimens from Ba Na and Kon Ha Nung are closely comparable (Table 2, Fig. 3).

**Table 2.** Leaf sizes.

**Locations**	**Specimens accession numbers**	**Leaf length (cm)**	**Leaf width (cm)**	**Leaf area (cm^2^)**
Kon Ha Nung (Gia Lai)	*Nguyen Tien Ban VN651* (VN0000039607	11.688	4.14	35.131
Kon Chu Rang (Gia Lai)	*DLUMag.131*	12.198	4.345	38.483
Ba Na (Da Nang)	*Poilane 7264* (VNM00000676)	11.889	4.073	33.866
Ba Na (Da Nang)	*Poilane 7264* (VNM00000678)	11.705	3.800	31.47
Peninsular Malaysia	*Haniff 4067* (K000681525)	20.138	7.571	113.391
Lomburr (Dam Rong, Lam Dong)	*DLUMag.126*	10.642	3.564	25.853

#### Stomatal apparatus

**Figure 4.**
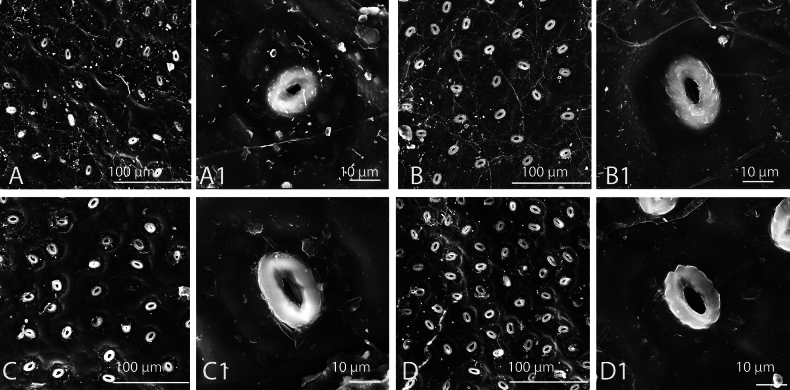
Stomatal characteristics of *M. praecalva* (**A–C**) and *M. tienii* (**D**). **A, A1**. *Poilane 7264* (VNM00000678); **B, B1**. *Nguyen Tien Ban VN651* (VN0000039607); **C, C1**. *DLUMag.131*; **D, D1**. *DLUMag.126*.

The stomatal apparatus on the abaxial epidermis was elliptic and distinctly raised. Although the stomatal dimensions were comparable to those of other specimens collected across Vietnam, the stomatal density was nearly double, reaching 473 stomata/mm^2^ (Fig. 4D, D1, Table 3).

**Table 3.** Stomatal data.

**Locations**	**Specimens accession numbers**	**Stomata length (SL, µm)**	**Stomata width (SW, µm)**	**Number of stomata per mm^2^ (№/mm^2^)**
Kon Ha Nung (Gia Lai)	*Nguyen Tien Ban VN651* (VN0000039607)	22.93 ± 1.61c	15.32 ± 1.66c	223.84 ± 33.02a
Kon Chu Rang (Gia Lai)	*DLUMag.131*	19.43 ± 3.67b	13.15 ± 2.72b	252.91 ± 12.33a
Ba Na (Da Nang)	*Poilane 7264* (VNM00000678)	16.52 ± 1.44a	9.75 ± 1.83a	222.38 ± 30.83a
Lomburr (Dam Rong, Lam Dong)	*DLUMag.126*	20.65 ± 1.57b	13.48 ± 0.95b	473.84 ± 43.89b

### Taxonomic treatment

#### Magnolia tienii

Taxon classificationPlantaeMagnolialesMagnoliaceae

H.T.Truong, N.T.A.Minh & T.V.Tran sp. nov.

4F4B4854-252B-5159-9DA3-597307D46184

urn:lsid:ipni.org:names:77387469-1

Figs 5, 6

##### Diagnosis.

*Magnolia tienii* is morphologically most similar to *M. praecalva* and *M. sinica* but differs from *M. praecalva* by having paired terminal flowers per twig (vs. solitary flowers), peduncles with two nodes on the first flower and 3–4 nodes on the second flower (vs. 3–4 nodes), elliptic leaves (vs. elliptic-oblong or oblanceolate leaves), and 11 tepals (vs. nine tepals).

**Figure 5.**
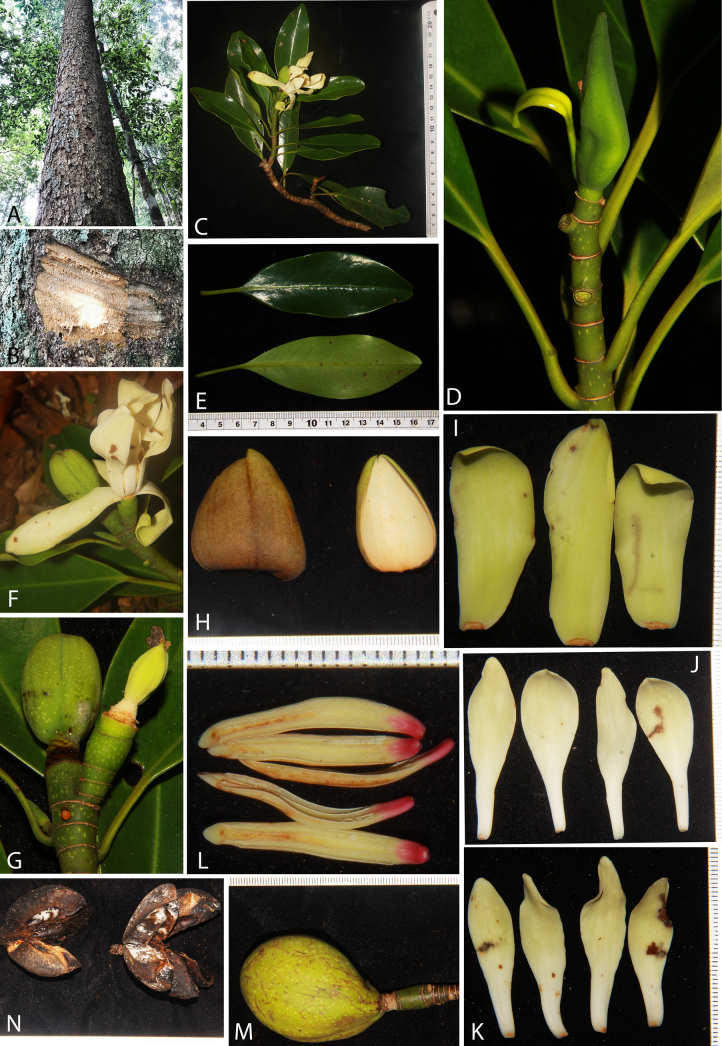
*Magnolia tienii* H.T.Truong, N.T.A.Minh & T.V.Tran. **A**. Twisted bole; **B**. Thick, fawn-colored bark; **C**. Flowering branch; **D**. Stipules free from the petiole; **E**. Leaf blade; **F, G**. Two flowers (fruits) borne terminally on the twigs; **H**. Spathaceous bracts; **I**. Outer tepals; **J**. Middle tepals; **K**. Inner tepals; **L**. Stamens; **M, N**. Fruit. Photographs by Hoang Thanh Truong from the type locality.

**Figure 6.**
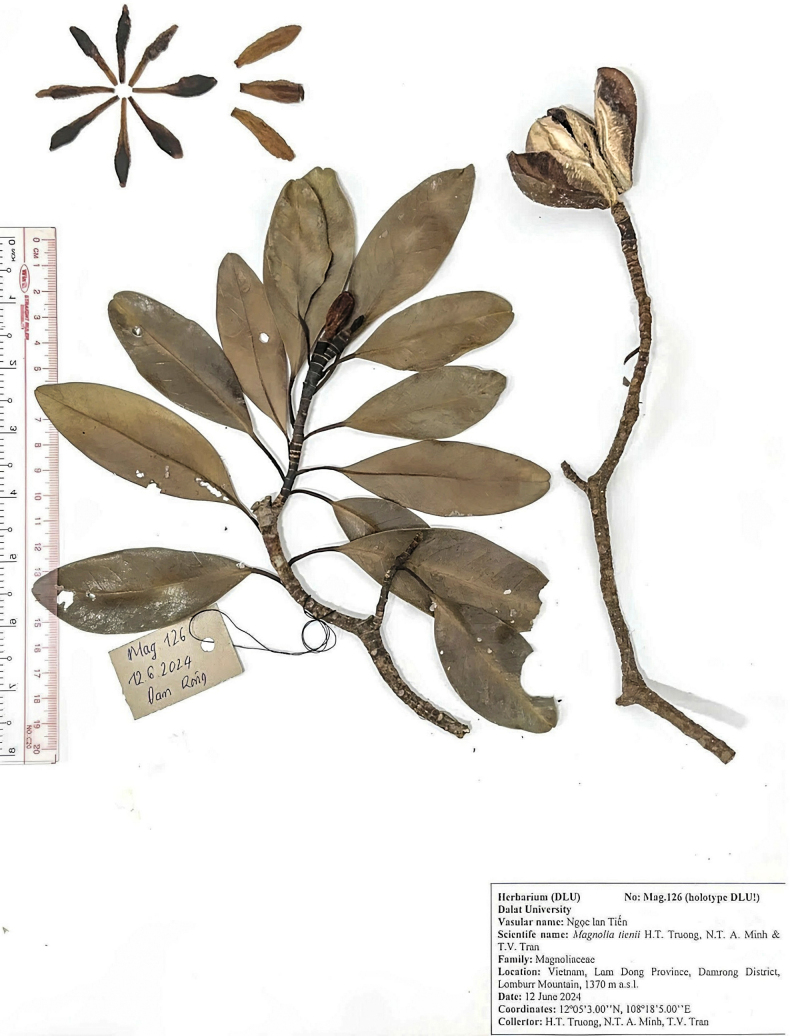
Holotype of *Magnolia tienii* H.T.Truong, N.T.A.Minh & T.V.Tran.

##### Type.

Vietnam • Lam Dong Province, Dam Rong District, Lomburr Mountain, 1370 m a.s.l. 12°05'3.00"N, 108°18'5.00"E, 12 June 2024, *H.T. Truong, N.T.A. Minh, T.V. Tran Mag.126* (holotype DLU!; isotype VNM!, VTN-Taynguyen Institute for Scientific Research!).

##### Etymology.

The new species is named in honor of Dr. Tran Van Tien for his contributions to *Magnolia* research.

##### Description.

Trees up to 20 m tall and up to 0.8 m diameter at breast height, glabrous throughout, with a straight bole. Bark pale brown to gray-brown, rugose to fissured. Old twigs brown, young twigs deep green, 4–6 mm in diameter. Stipules free from the petiole. Leaves spirally arranged; petiole 2–2.5 cm, without a stipular scar, base slightly enlarged; leaf blade elliptic, base gradually narrowly cuneate and decurrent on petiole, apex slightly-attenuate or obtuse, concave, and often emarginate, 9.5–10.5 × 3.0–3.5 cm, leathery, abaxially pale green, adaxially deep green and glossy; midvein prominent on both surfaces, secondary veins 10–12 on each side of midvein, meeting in an intramarginal vein not close to the margin, reticulate veins sparse and prominent on both surfaces when dry. Peduncle 0.4–0.6 cm, stout, 1–2 nodes (first flower) and 3–4 nodes (second flower). Two flowers terminal on the twigs. Spathaceous bracts just next to tepals, green-brown, obovoid to ovoid, caducous, apex concave. Tepals 11, in three whorls, cream white, fragrant; three outer tepals oblong-spatulate, 4–4.5 × 1.2–1.5 cm, apex obtuse; eight middle and inner tepals obovate-spatulate, 3–3.5 × 1–1.2 cm, apex obtuse or acute. Stamens ca. 45; connective appendage acute, narrowly triangular; anthers dehiscent introrsely, 1.5–1.6 × 0.1–0.2 cm, base reddish. Gynoecium long ovoid, receptacle short, carpels attached at approximately the same height, entirely hidden within androecium; carpels 2–4; ovules 2–3 per carpel. Fruiting peduncles stout, glabrous, 1–1.2 × 0.4–0.6 cm thick. Fruits green-brown when mature, dark brown when dry, obovoid, base cuneate, apex globose, 5–8.5 × 3.5–6.5 cm; mature carpels narrowly long ellipsoid to obovoid-ellipsoid, 4.5–5.5 × 3.8–4.2 cm, thickly woody, dehiscing completely along ventral sutures, abaxially with coarse lenticels, splitting into 2–4 valves, late the carpels more separating from each other, in the center a columella persistent with the attached seeds. Scars of perianth and stamens a long receptacle under fruit 5–6 mm, concave. Seeds 1–3 per carpel.

##### Phenology.

The plants were found flowering from May to June and fruiting from June to July.

##### Distribution and habitat.

*Magnolia tienii* grows scattered in primary forest at elevations of 1,300–1,400 m a.s.l. on Lomburr Mountain, Dam Rong District, Lam Dong Province. In contrast, historical specimens of allied taxa collected from other regions occur at lower elevations, typically below 1,000 m a.s.l

##### Preliminary conservation status.

*Magnolia tienii* is currently known only from a single localized population on Lomburr Mountain (Dam Rong District, Lam Dong Province, Vietnam). An extensive field survey within the area revealed fewer than five mature individuals growing in primary forest, with an estimated area of occupancy (AOO) of less than 2 ha. Consequently, following the IUCN Red List Categories and Criteria (IUCN 2024), *M. tienii* is provisionally assessed here as Critically Endangered (CR D) because of its critically small population size (fewer than 50 mature individuals). Additional field investigations are strongly recommended to monitor this population and identify potential additional localities.

### Target amplification and phylogeny construction

**Figure 7.**
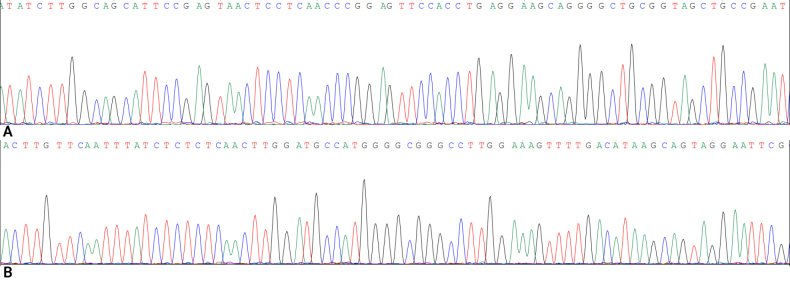
Representative fragments of the *rbcL* gene sequencing chromatograms. **A**. Forward sequence; **B**. Reverse sequence.

The samples collected from Lomburr Mountain were subjected to molecular identification and authentication. PCR amplification of the *rbcL* region was successfully achieved, and the resulting amplicons were sequenced using the Sanger method (Fig. 7). The obtained chromatograms exhibited high-quality, well-defined, non-overlapping peaks for both strands of the target gene, with minimal background noise and no ambiguous bases, thereby confirming the reliability of the sequencing data. Subsequently, the low-quality terminal regions of both sequences were trimmed before phylogenetic analysis.

**Figure 8.**
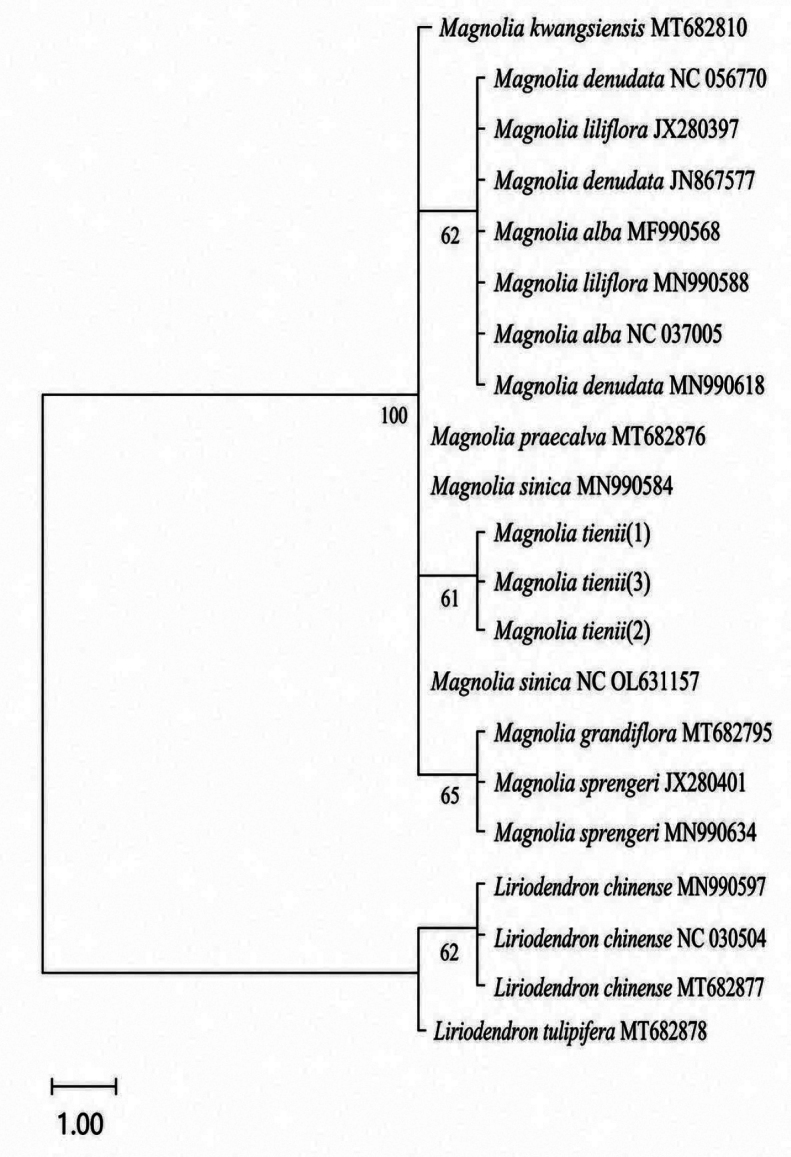
Phylogenetic relationships between *M. tienii* and its allies based on *rbcL* sequence data. Bootstrap values from 1,000 replicates are indicated above the nodes.

The MP-based phylogenetic tree is presented in Fig. 8. The analysis included 21 nucleotide sequences. There were 526 positions in the final dataset. Phylogenetic analyses supported a close relationship between *Magnolia tienii* and other species of *Magnolia*. The *rbcL* dataset comprised 14 taxa belonging to *Magnolia* and four taxa belonging to the genus *Liriodendron*, which served as the outgroup. All *M. tienii* samples formed a monophyletic group with *M. sinica* and *M. praecalva*, with a bootstrap value of 40, suggesting genetic divergence and supporting its recognition as a potentially independent evolutionary lineage.

The 526-bp *rbcL* sequences of *M. tienii* were compared with those of *M. sinica* and *M. praecalva* by pairwise alignment. The results revealed a single nucleotide difference between *M. tienii* and each of the two reference species, corresponding to 99.81% sequence identity (525/526 bp). Specifically, at this polymorphic site near the 3' end of the amplified fragment, *M. tienii* possesses the codon CGC, whereas both *M. sinica* and *M. praecalva* contain CGT. This substitution occurs at the third position of the codon and is synonymous, encoding arginine (Arg) in all three taxa. No insertions or deletions were detected within the aligned region, and the predicted amino acid sequence of the RbcL protein was identical among the three species.

Although molecular evidence suggested that *M. tienii* may represent a novel species, phylogenetic data alone were insufficient for formal species delimitation. Therefore, a comprehensive comparative analysis was essential to evaluate *M. tienii* against *M. sinica* and *M. praecalva*, with a focus on key vegetative and reproductive macromorphological characters, including leaves, flowers, and fruits. Furthermore, the inclusion of additional molecular markers and broader taxon sampling in future studies may enhance phylogenetic resolution and provide robust support for defining these taxonomic boundaries.

## Discussion

In the phylogenetic analysis, *M. praecalva*, *M. sinica*, and *M. tienii* formed a distinct clade, indicating a close evolutionary relationship among the three species. The *rbcL* gene is widely recognized as a highly conserved marker in angiosperms, including the genus *Magnolia*, and consequently exhibits limited sequence variability among closely allied taxa. In the present study, *Magnolia tienii* exhibited only a single nucleotide substitution relative to both *M. sinica* and *M. praecalva* within the 526-bp *rbcL* fragment. This substitution occurred at the third codon position and was synonymous, resulting in no change in the amino acid sequence of the RbcL protein. This single synonymous mutation suggests that the observed variation reflects a neutral nucleotide polymorphism rather than functional divergence at the protein level. This evolutionary conservation aligns with their shared macromorphological framework, as members of this section possess stipules free from the petiole and lack a stipular scar (Dandy 1933; Nooteboom 1985; Xia et al. 2008). Furthermore, substantial morphological differentiation in leaf morphology, floral structures, and tepal number remains critical for delineating species boundaries within *Magnolia* (Nooteboom 2012; Duy et al. 2015; Vu et al. 2015). Morphological comparisons (Table 4, Fig. 3) demonstrate that *M. tienii* clearly differs from its closest relatives in several vegetative and reproductive traits. Although environmental conditions can influence leaf phenotypes, such plastic effects are generally more pronounced in quantitative traits, whereas qualitative characters tend to remain evolutionarily stable (Tucker 1964; Baranova 1972; Metcalfe and Chalk 1979; Hu et al. 2018). In this study, both qualitative and quantitative traits robustly support the distinctiveness of the new species. Specifically, qualitative characters such as paired terminal flowers and an 11-tepalate perianth, together with quantitative differences in leaf area and stomatal density, distinguish *M. tienii* from its allied congeners.

**Table 4.** Morphological comparison of *Magnolia tienii* H.T.Truong, N.T.A.Minh & T.V.Tran, sp. nov. with *Magnolia praecalva* (Dandy) Figlar & Noot. and *Magnolia sinica* (Y.W.Law) Figlar & Noot.

**Characters**			***M. praecalva***	***M. sinica***	***M. tienii***
Leaves	Petiole		2–3 cm long	1.5–2 cm long	2–2.5 cm long
	Leaf blade		Elliptic-oblong or oblanceolate, 7–20 × 3–3.5	Narrowly obovate to narrowly obovate-elliptic, 15–26(–30) × 5–8(–9.5) cm	Elliptic, 9.5–10.5 × 3.0–3.5 cm
		Apex	Obtuse to rounded	Rounded and with a ca. 5 mm acute tip	Slightly attenuate or obtuse
		Base	Cuneate or attenuate-cuneate	Gradually narrowly cuneate	Cuneate or attenuate-cuneate
Flowers			Solitary	Solitary	Two flowers
			Peduncle with 3–4 nodes	Peduncle with 3–4 nodes	1–2 nodes (first flower) and 3–4 nodes (second flower) of peduncle
			Nine tepals	Nine tepals	11 tepals
Fruit		Shape and size	Subglobose, 3.5–6 × 3.5–6 cm	Obovoid to ellipsoid-ovoid, 5–8.5 × 3.5–6.5 cm	Obovoid or, base cuneate apex globose, 5–8.5 × 3.5–6.5 cm
		Peduncle	0.5–20 mm long	Ca. 10 mm long	10–12 mm long

## Supplementary Material

XML Treatment for Magnolia tienii

## Additional information

### Artificial Intelligence (AI) use

The authors accept full responsibility for the content of the manuscript, including the disclosure of any use of AI.

No AI tools were used in the preparation of this manuscript.

### Author ORCIDs

N.T.A. Minh https://orcid.org/0009-0000-7521-2065

T.T. Vinh https://orcid.org/0000-0001-6410-1423

T.T.L. Anh https://orcid.org/0009-0006-3042-9168

N.H. Nghia https://orcid.org/0009-0002-7936-8029

H.T. Truong https://orcid.org/0000-0002-8799-5818

L.D. Thuan https://orcid.org/0000-0002-6459-3058

T.V. Tien https://orcid.org/0000-0001-6539-0327

### Data availability

All of the data that support the findings of this study are available in the main text.
